# Endocervical Polyp as a Cause of Genital Bleeding in the Second Half of Pregnancy: A Case Report

**DOI:** 10.7759/cureus.74036

**Published:** 2024-11-19

**Authors:** Rodolfo Arturo de la Trinidad Torres Aray, Isbelia I Aray Gonzalez

**Affiliations:** 1 Research, Universidad de Buenos Aires, Buenos Aires, ARG; 2 Obstetrics and Gynecology, Universidad de Carabobo, Maracay, VEN

**Keywords:** cervical polyp, gestational bleeding, pregnancy, pregnancy complication, vaginal bleeding

## Abstract

Cervical polyps are typically benign exophytic lesions that are often asymptomatic and small during pregnancy. However, they can occasionally cause significant symptoms, leading to gestational complications. A lack of awareness of this condition, combined with inadequate diagnosis and treatment, can pose risks to maternal and fetal health, potentially resulting in complications or pregnancy loss. This case report describes a 36-year-old primigravida at 23 weeks of gestation who presented with sudden vaginal bleeding. Physical examination revealed an exophytic cervical lesion, which was successfully treated with polypectomy, allowing the pregnancy to progress to term without further bleeding. This case underscores the importance of promptly diagnosing the etiology of vaginal bleeding in the second half of pregnancy and considering endocervical polyps as a potential cause, emphasizing early diagnosis and intervention to reduce maternal and fetal risks.

## Introduction

Cervical polyps are predominantly benign and asymptomatic lesions commonly found during routine physical exams in outpatient settings [1]. However, during pregnancy, they gain particular significance due to their potential to cause gestational complications. While cervical polyps can occur at any stage of pregnancy, their exact prevalence in this population remains unknown [1].

In symptomatic pregnant individuals, cervical polyps can present with bleeding, postcoital bleeding, vaginal discharge, cervical infection, and even symptoms resembling threatened preterm labor [2]. Asymptomatic cases are often managed conservatively [1], but the mere presence of these lesions increases the risk of late spontaneous abortion, preterm birth, or cervical insufficiency [3]. It is suggested that ascending infection and/or inflammation may be mechanisms through which cervical polyps contribute to these complications [4].

In this report, we present the case of a pregnant patient in the second half of pregnancy (defined as after 22 weeks of gestation) with a symptomatic cervical polyp presenting with bleeding [5]. This case required prompt pathology assessment to rule out placental bleeding and prevent complications associated with the identified lesion, ultimately reducing maternal and fetal morbidity and mortality.

## Case presentation

A 36-year-old primigravida patient with a medical history of vaginismus, at 23+6 weeks of gestation, presented to the outpatient gynecology clinic with a history of recent-onset heavy vaginal bleeding, without accompanying signs or symptoms. The patient had received prenatal care since the beginning of her pregnancy at the same clinic but had been unable to undergo a Pap smear or cervical examination due to her history of vaginismus.

On physical examination, the patient was in good general condition, alert, and oriented to person, place, and time, without lower limb edema, and with vital signs within normal ranges. Uterine height was 20 cm, consistent with the gestational age, fetal heart rate was 143 beats per minute, and the fetus was in cephalic presentation.

**Figure 1:**
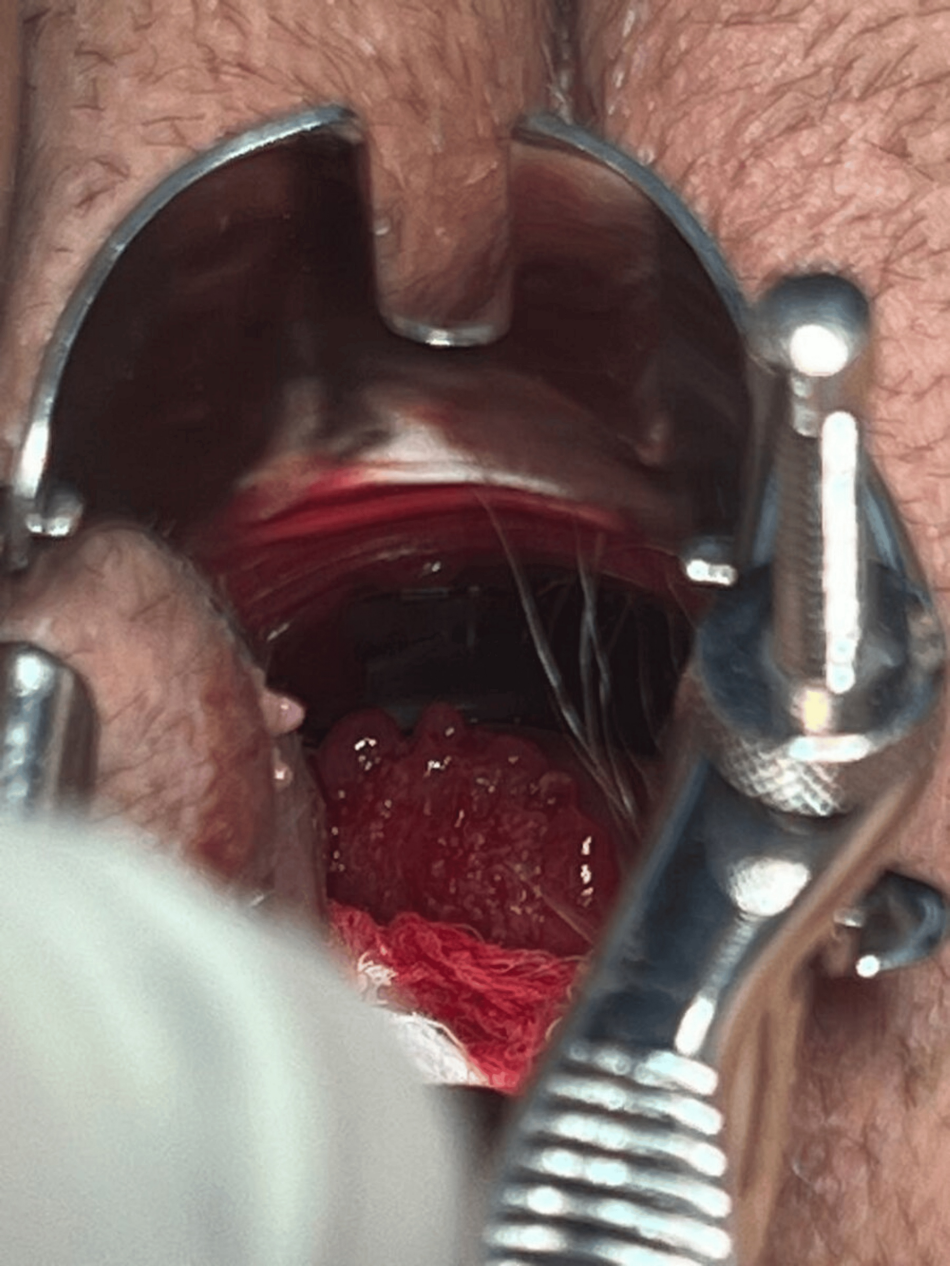
Gross image of the cervical exophytic lesion

Due to the inability to perform a speculum examination because of vaginismus, the patient was sedated to complete the physical exam. No vaginal septa or deformities were observed, but a friable, bleeding, reddish macroscopic lesion was visualized at the cervical level, obstructing the view of the cervical os (Figure 1).

The lesion was excised using Foster forceps, and a cervical biopsy was taken and sent to pathology for histopathological analysis. The results confirmed the presence of a benign endocervical polyp. Following the excision of the exophytic lesion, the patient did not experience further episodes of vaginal bleeding or other complications throughout the pregnancy, which culminated in a term birth at 37 weeks + 3 days via segmental cesarean section. During the surgical procedure, no macroscopic exophytic lesions were found upon exploration of the uterine cavity.

## Discussion

This case report underscores the importance of considering endocervical polyps as a potential cause of vaginal bleeding in the second half of pregnancy. Although generally benign and asymptomatic, these lesions can occasionally lead to significant complications, as seen in this case, where bleeding necessitated surgical intervention. The successful management of the patient through polypectomy allowed the pregnancy to continue without further issues, highlighting the safety of this approach when clinically indicated.

Endocervical polyps are relatively rare during pregnancy and are often incidental findings in asymptomatic women during routine gynecological exams [1]. However, when symptoms such as vaginal bleeding occur, careful evaluation is crucial to avoid severe complications [6]. Vaginal bleeding during pregnancy is a concerning symptom, with numerous potential causes, including placental abruption, placenta previa, infections, and cervical disorders [7]. In such cases, endocervical polyps should be included in the differential diagnosis, especially after more common and potentially serious conditions are excluded.

Polypectomy, as performed in this case, proved effective in halting the bleeding without compromising the pregnancy. However, surgical intervention in pregnant patients must be approached cautiously, weighing the benefits of resection against the risks, such as uterine contractions or postoperative infections. While conservative management is a valid treatment option for asymptomatic polyps [8], early intervention in symptomatic cases, as demonstrated here, can prevent complications like preterm labor or cervical insufficiency [1].

Vaginismus, a significant factor in this case, complicated the initial evaluation, delaying routine cervical sampling and a full assessment of the cervical canal. This highlights the need for tailored diagnostic strategies to meet the individual needs of each patient, ensuring that no underlying pathology is overlooked [9]. The use of sedation facilitated the necessary evaluation and treatment, ensuring the safety of both the mother and the fetus.

The potential mechanisms by which endocervical polyps complicate pregnancy include chronic inflammation and ascending infection, which may contribute to cervical insufficiency or preterm birth [10]. Although no active infections or inflammations were observed in this case, the presence of a bleeding cervical lesion required intervention to mitigate the risk of further complications. This case emphasizes the importance of early diagnosis and individualized management, as untreated cases could lead to preventable complications.

## Conclusions

Endocervical polyps, although rare during pregnancy, can unexpectedly cause vaginal bleeding in the second half of gestation, requiring careful clinical management. In this case, prompt intervention helped prevent major complications and allowed the pregnancy to continue to term. This report emphasizes the importance of a thorough evaluation in any case of vaginal bleeding during pregnancy, even when more common causes have been ruled out. It also highlights the need to tailor gynecological procedures to the individual characteristics of each patient, as demonstrated in this case, where vaginismus complicated the initial diagnosis. A personalized approach to managing such cases can improve maternal-fetal outcomes and should be prioritized in clinical practice.
